# Supplementary material for: Tropical precipitation response to anthropogenic climate change in recent decades
Source: Nat Commun. 2026 Mar 26;17:4450. doi: 10.1038/s41467-026-71187-4 (PMC13183973; doi:10.1038/s41467-026-71187-4)
Supplement: Supplementary file 1 — Supplementary Information [file 41467_2026_71187_MOESM1_ESM.pdf]

Supplement for 'Tropical Precipitation Response to Anthropogenic Climate Change in Recent Decades'

Ligin Joseph[1*†], Pascal Terray[2†], K.P. Sooraj[3], Sébastien Masson[2]

[1]School of Ocean and Earth Science, University of Southampton, Southampton, United Kingdom.
[2]Sorbonne Universités (UPMC, Univ Paris 06)-CNRS-IRD-MNHN, LOCEAN Laboratory, 4 place Jussieu, Paris, France.
[3]Centre for Climate Change Research, Indian Institute of Tropical Meteorology, Ministry of Earth Sciences, Pune, India.

*Corresponding author(s). E-mail(s): l.joseph@soton.ac.uk;
†These authors contributed equally to this work.

1 Materials

The monthly precipitation data are obtained from the Global Precipitation Climatology Project (GPCP) version 2.3 [1], which provides merged satellite and gauge-based estimates on a global scale. Monthly air temperature data are sourced from the Berkeley Earth Temperature dataset [2], combined with air temperature over sea ice regions. Sea surface temperature data are taken from the NOAA Optimum Interpolation SST (OISST) version 2 [3], which blends satellite and in situ measurements. The 850-hPa and 200-hPa wind fields are obtained from the NCEP–DOE Reanalysis 2 (NCEP2) [4], and the 200-hPa velocity potential fields are computed from the 200-hPa winds using the Windspharm Python package[5]. All datasets are analyzed for the period 1979–2024, ensuring consistency with the satellite era and ERA5 used in the main text.

2 Biases in the coupled models

The climatological biases in precipitation, surface temperature, and 850-hPa winds in the ctrl configuration of the CFS and SINTEX models are shown in Supplementary Figure 9. These two models exhibit systematic errors similar to CMIP models in simulating tropical SSTs, winds,and precipitation (see Terray et al. [6] and Supplementary

Figure 2b). Both models show the double-ITCZ precipitation bias, which is a long-standing issue across the past three phases of CMIP models [7–9]. In addition, both models show a wet bias in the Indo-Pacific warm pool and equatorial Atlantic, and dry biases over the Indian subcontinent, South America, and the central and eastern equatorial Pacific. CFS additionally shows a widespread wet bias across much of the Indian Ocean, whereas SINTEX displays a wet bias north of the equator in the Indian Ocean and a dry bias in the southeastern equatorial Indian Ocean.

Similar to the precipitation bias, the lower-tropospheric circulation exhibits broadly similar bias patterns in both models. Several of these biases, including weakened or easterly wind biases in the Southern Hemisphere mid-latitudes and trade-wind biases over the tropical Pacific, are also well-documented features of CMIP-class models [7–10].

Unlike precipitation and circulation biases, for which the two models exhibit broadly similar spatial patterns, surface temperature biases differ substantially between the models. The CFS model shows a pronounced cold bias across much of the Northern Hemisphere, especially over land. This severe cold bias is partly corrected in the MODIS-ctrl configuration of CFS (see Terray et al. [6]). In contrast, the surface temperature bias in the SINTEX model is smaller in magnitude overall, but a significant cold bias is still present in the northern high latitudes and the arid regions of the Sahara, Arabia and Middle East. Again, this cold bias is reduced in the MODIS-ctrl configuration of SINTEX [6]. Both models also exhibit a warm bias over the Southern Hemisphere polar regions and the southeastern Pacific and Atlantic Oceans as most of the CMIP models.

All these similarities with the CMIP models suggest that the results obtained with the CFS and SINTEX models are also pertinent for CMIP models.

3 Decomposition of observed tropical precipitation change into physical components

To better understand the physical drivers of observed precipitation changes, we apply a moisture budget decomposition framework adapted from Chadwick et al. [11], extended here to ERA5 reanalysis data. This method separates the total precipitation change (defined as the difference between the 2005–2024 and 1981–2000 means) into several physically interpretable components. The decomposition is expressed as:

$$\begin{aligned}\Delta P = M^{*}\Delta q_{\mathrm{CC}} + M^{*}\Delta q_{\mathrm{RH}} + q_{\mathrm{CC}}\Delta M^{*}_{\mathrm{div}} \\ + q_{\mathrm{CC}}\Delta M^{*}_{\mathrm{spat}} + \Delta q_{\mathrm{CC}}\Delta M^{*}\end{aligned} \tag{1}$$

Each term in Eq. 1 has a clear physical interpretation:

- $M^{*}\Delta q_{\mathrm{CC}}$: The *thermodynamic component*, representing precipitation increases due to warming-induced increases in specific humidity under fixed relative humidity (Clausius–Clapeyron component). The vertical mass flux is defined as $M^{*} = P/q$, where P is the mean precipitation and q is the mean near-surface specific humidity. Δq_{CC} is the change in 2-m specific humidity expected under fixed relative humidity,

calculated using the local 2-m temperature change and the August–Roche–Magnus formulation of the Clausius-Clapeyron relationship.

- $M^*\Delta q_{\mathrm{RH}}$: The contribution from changes in *near-surface relative humidity*, calculated as the residual between total specific humidity change and the Clausius–Clapeyron component.
- $q_{\mathrm{CC}}\Delta M^*_{\mathrm{div}}$: The contribution from *changes in the magnitude of upward vertical mass flux due to large-scale circulation changes* (i.e., divergence feedback). It is computed as the spatial mean of ΔM^* and approximated via a scaling coefficient α, defined as

$$\alpha = -\frac{\Delta M^*}{M^*_{\mathrm{early}} \cdot \Delta T},$$

where M^*_{early} is the baseline tropical-mean mass flux and ΔT is the tropical mean near-surface temperature change. The divergence component is then estimated as

$$\Delta M^*_{\mathrm{div}} = -\alpha \cdot M^*_{\mathrm{mean}}.$$

where M^*_{mean} is the time mean of M^*.

- $q_{\mathrm{CC}}\Delta M^*_{\mathrm{spat}}$: The dynamic component due to *spatial shifts in the pattern of convection.* It is obtained by subtracting the divergence-related change from the total change in mass flux: $\Delta M^*_{\mathrm{spat}} = \Delta M^* - \Delta M^*_{\mathrm{div}}$.
- $\Delta q_{\mathrm{CC}}\Delta M^*$: A *nonlinear interaction term* between moisture and circulation changes.

All terms are expressed in mm day^{-1} and computed over the tropical belt (30°S–30°N). Here, the symbol Δ denotes the difference between the recent period (2005–2024) and the baseline period (1981–2000). Further methodological details are available in Chadwick et al. [11].

Figure 5 shows the observed precipitation changes between the two periods, together with the contributions from different components derived using equation 1. First, the spatial pattern of tropical precipitation changes (Fig 5a) is characterized by increased precipitation north of the ITCZ in the Pacific and Atlantic Oceans, enhanced rainfall over the Indo-Pacific warm pool, and reduced precipitation south of the ITCZ in the tropical Pacific and closely resembles the long-term precipitation trends (Supplementary Figure 1a) . Consistent with the simple moisture budget analysis presented in the main text (see Section 2.2 and Supplementary Figure 2), this decomposition indicates that the dynamic component (Supplementary Figure 5b) is the dominant contributor to the observed precipitation changes, whereas the thermodynamic component plays a comparatively minor role. Other terms, including contributions from changes in near-surface relative humidity, circulation weakening via divergence feedback, the combined thermodynamic and divergence feedback terms, and the nonlinear interaction term, make only small contributions relative to the dynamic component.

These results further confirm that shifts in atmospheric circulation patterns are the primary driver of the observed precipitation changes. The physical mechanisms responsible for these circulation changes are investigated in detail in the main text.

Finally, Supplementary Figure 5h shows that the residual between the observed precipitation changes and the reconstructed changes—obtained as the sum of all decomposed components—is minimal, demonstrating the robustness of the reconstruction.

4 Supplementary Table and Figures

Supplementary Table 1 List of CMIP6 models and institutions used in this study.

Model Name	Institution
ACCESS-CM2	CSIRO-ARCCSS
ACCESS-ESM1-5	CSIRO
BCC-CSM2-MR	BCC
CAMS-CSM1-0	CAMS
CESM2-WACCM	NCAR
CESM2	NCAR
CMCC-CM2-SR5	CMCC
CNRM-CM6-1-HR	CNRM-CERFACS
CNRM-CM6-1	CNRM-CERFACS
CNRM-ESM2-1	CNRM-CERFACS
CanESM5	CCCma
EC-Earth3-Veg-LR	EC-Earth-Consortium
EC-Earth3-Veg	EC-Earth-Consortium
EC-Earth3	EC-Earth-Consortium
FGOALS-g3	CAS
GFDL-CM4	NOAA-GFDL
GFDL-ESM4	NOAA-GFDL
HadGEM3-GC31-LL	MOHC
IITM-ESM	CCCR-IITM
INM-CM4-8	INM
INM-CM5-0	INM
IPSL-CM6A-LR	IPSL
KACE-1-0-G	NIMS-KMA
KIOST-ESM	KIOST
MIROC-ES2L	MIROC
MIROC6	MIROC
MPI-ESM1-2-HR	MPI-M
MPI-ESM1-2-LR	MPI-M

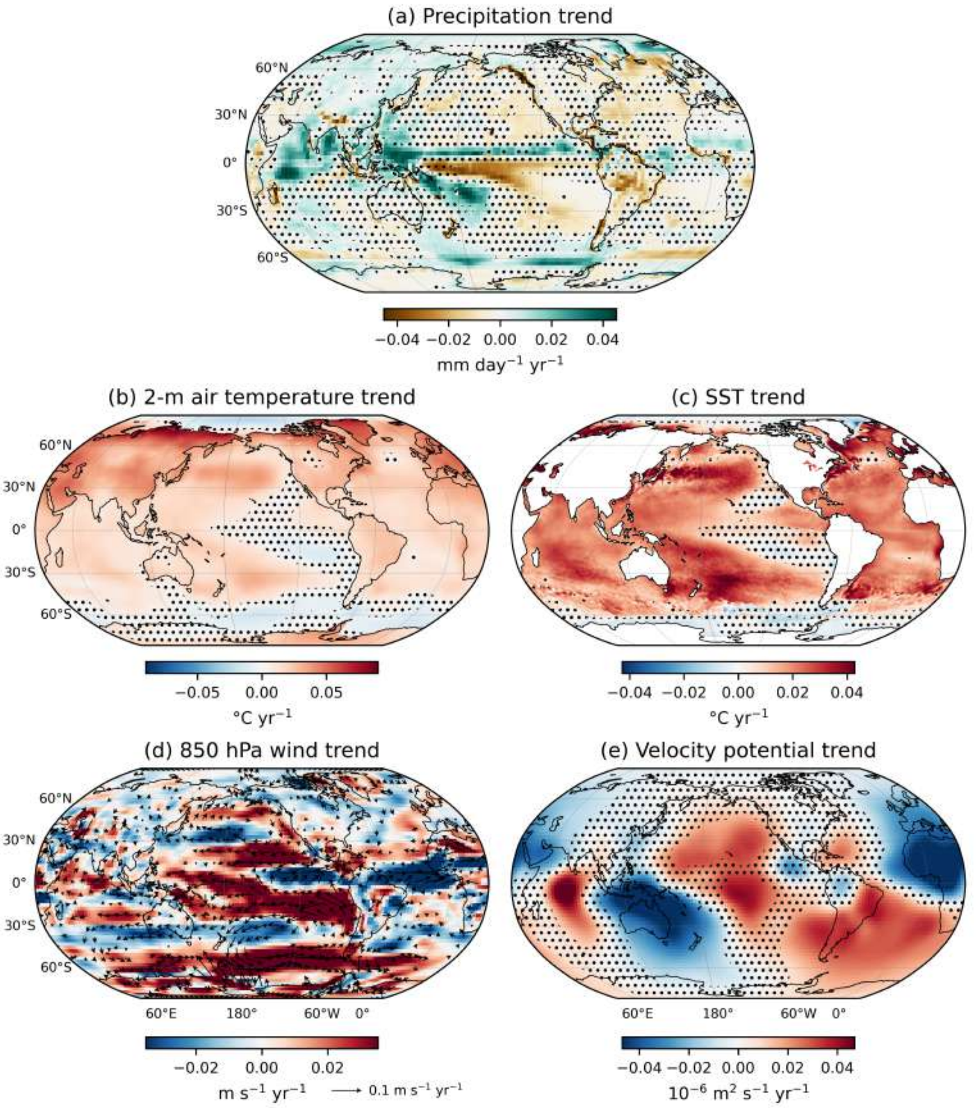

Supplementary Figure 1 Global climate trends (1979-2024) in (a) precipitation (GPCP; mm day^{-1} yr^{-1}), (b) 2-meter air temperature (Berkeley Earth; °C yr^{-1}), (c) sea surface temperature (OISST; °C yr^{-1}), (d) 850-hPa wind vectors (arrows) and speed (shading; m s^{-1} yr^{-1}) from NCEP2, and (e) 200-hPa velocity potential (NCEP2; m^2 s^{-1} yr^{-1}). Dots represent regions with statistically insignificant trends at 90% confidence level. Wind vectors in panel (d) are plotted only if at least one component of the wind trend is statistically significant at 90% confidence level.

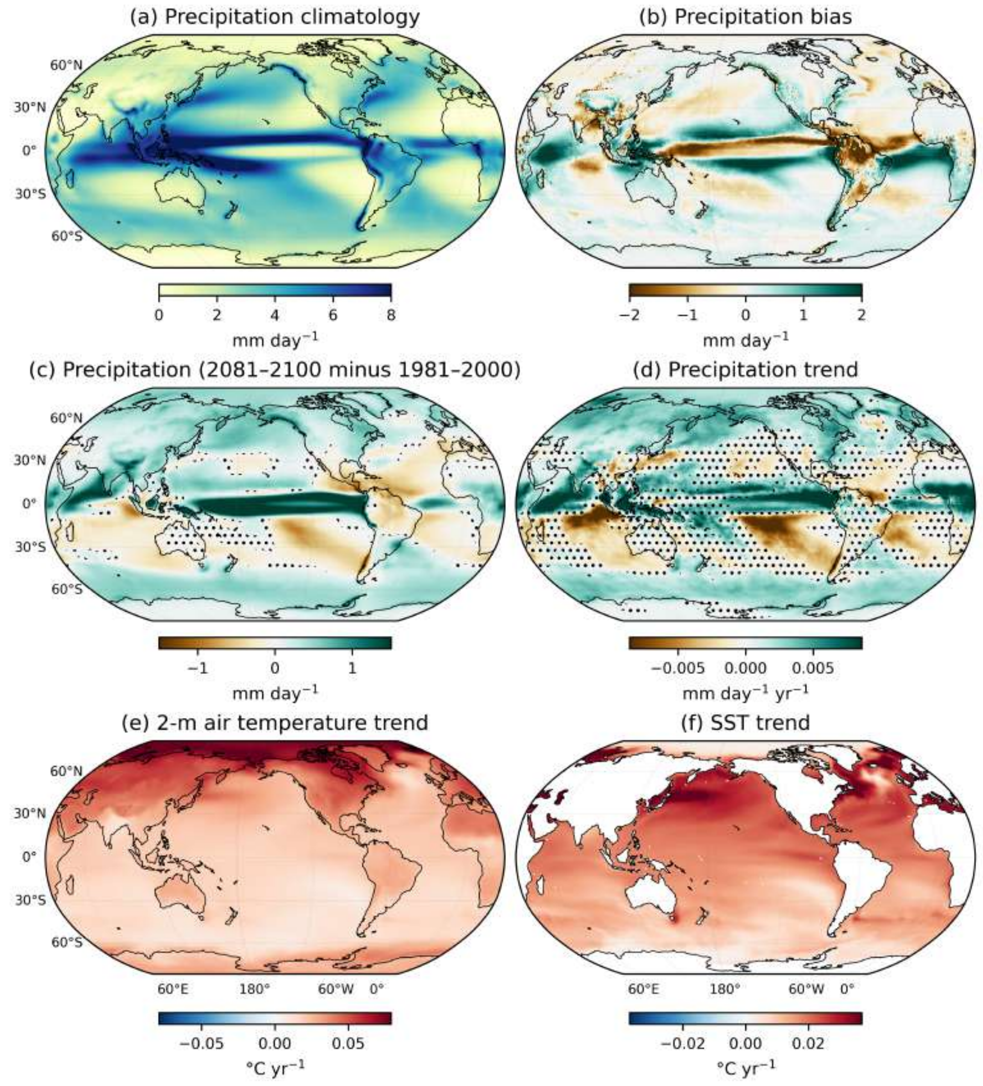

Supplementary Figure 2 Climatology, bias, future changes, and trends in CMIP6 models in (a) precipitation MME climatology computed from the 1979-2014 period from CMIP6 historical simulations (mm day^{-1}), (b) precipitation bias in CMIP6 MME compared to ERA5 climatology during the 1979-2014 period (mm day^{-1}), (c) projected future changes in precipitation (2081-2100 (SSP585) minus 1981-2000 (historical)) from CMIP6 simulations and projections (mm day^{-1}), (d) precipitation trend (mm day^{-1} yr^{-1}), (e) 2-m air temperature trend (°C yr^{-1}), and (f) SST trend (°C yr^{-1}). In panels (d), (e) and (f), the trends are computed over the 1979-2024 period combining CMIP6 historical and SSP245 simulations. Dots represent regions with statistically insignificant trends at 90% confidence level.

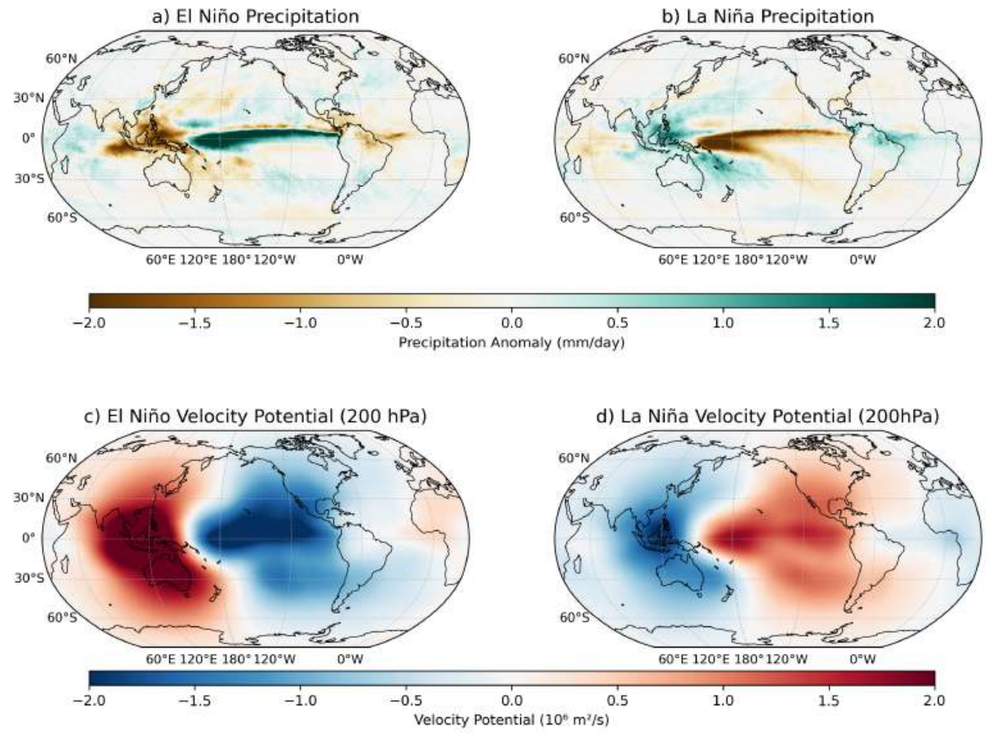

Supplementary Figure 3 Composite maps during El Niño and La Niña years for precipitation (a,b; mm day^{-1}) and 200-hPa velocity potential anomalies (c,d; 10^{-6} m^2 s^{-1}) during (a,c) El Niño and (b,d) La Niña years. All the data are from ERA5. El Niño years include: 1982, 1987, 1991, 1992, 1997, 2002, 2015, 2019, 2023, and La Niña include: 1981, 1984, 1985, 1988, 1989, 1996, 1999, 2000, 2007, 2008, 2011, 2021, 2022.

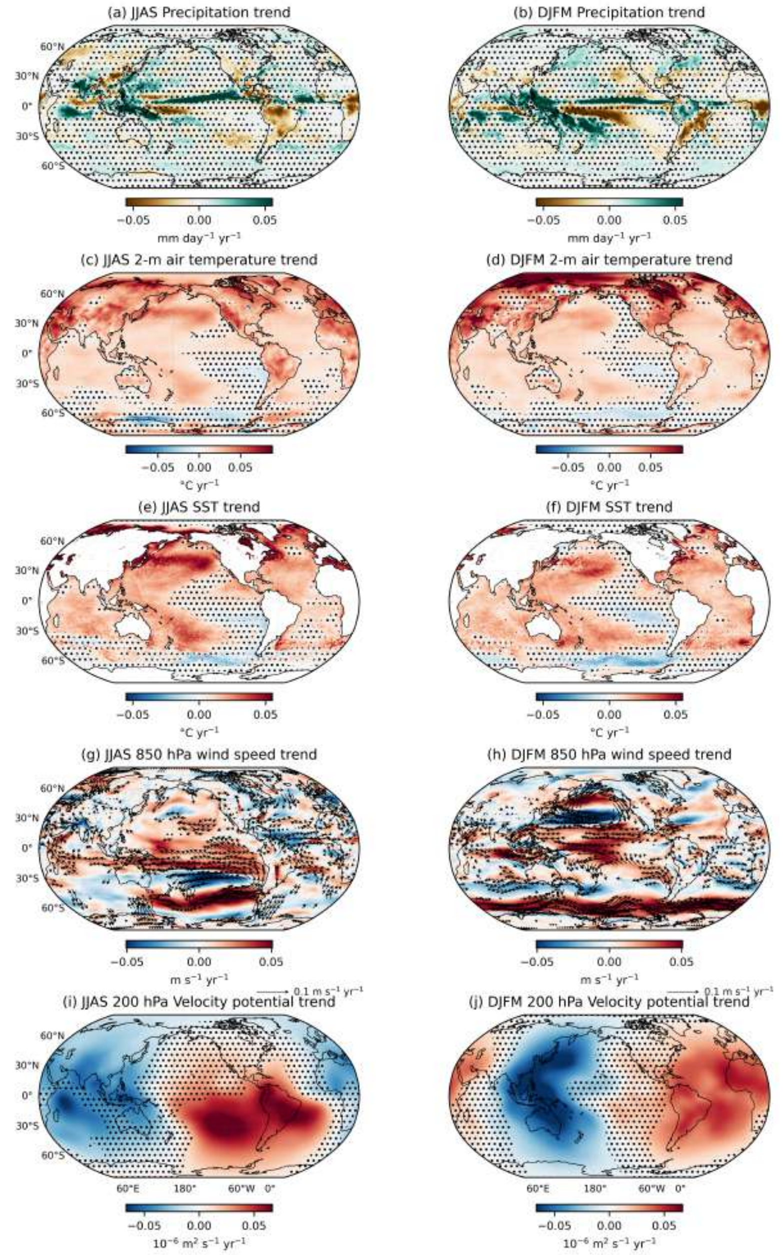

Supplementary Figure 4 Seasonal climate trends (1979–2024). Left column: boreal summer (JJAS); right column: boreal winter (DJFM). Shading shows linear trends per year for (a,b) precipitation (GPCP; mm day^{-1} yr^{-1}), (c,d) 2-meter air temperature (Berkeley Earth; °C yr^{-1}), (e,f) sea-surface temperature (OISST; °C yr^{-1}), (g,h) 850-hPa wind speed with wind-vector trends overlaid (NCEP2; m s^{-1} yr^{-1}), and (i,j) 200-hPa velocity potential (NCEP2; 10^{-6} m^2 s^{-1} yr^{-1}). Dots mark grid points where the trend is not significant at 90% confidence level. Arrows in (g,h) denote trends in the 850-hPa wind vectors and are drawn only where at least one wind component is significant at the 90% confidence level.

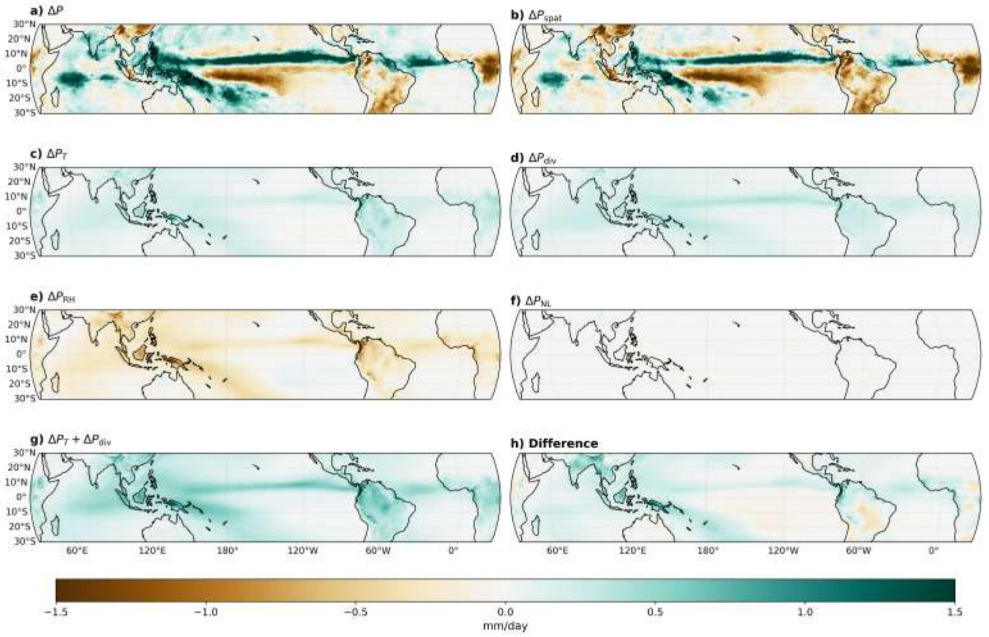

Supplementary Figure 5 Components of tropical precipitation change The left column shows (from top to bottom): (a) the total observed change in precipitation (ΔP, 2005-2024 minus 1981-2000), (c) the thermodynamic component related to Clausius–Clapeyron scaling under fixed relative humidity (ΔP_T), (e) the contribution from near-surface relative humidity change (ΔP_{RH}), and (g) the sum of the thermodynamic and divergence feedback components ($\Delta P_T + \Delta P_{\text{div}}$). The right column shows (from top to bottom): (b) the spatial shift component associated with changes in the location of convection (ΔP_{spat}), (d) the dynamic weakening of circulation via divergence feedback (ΔP_{div}), (f) the nonlinear interaction term (ΔP_{NL}), and (h) the residual between the observed ΔP and the sum of all reconstructed components. All values are in mm/day and are plotted for the tropical belt (30°S–30°N) using a consistent color scale. The decomposition follows the framework of ref [11], extended here to observational reanalysis data.

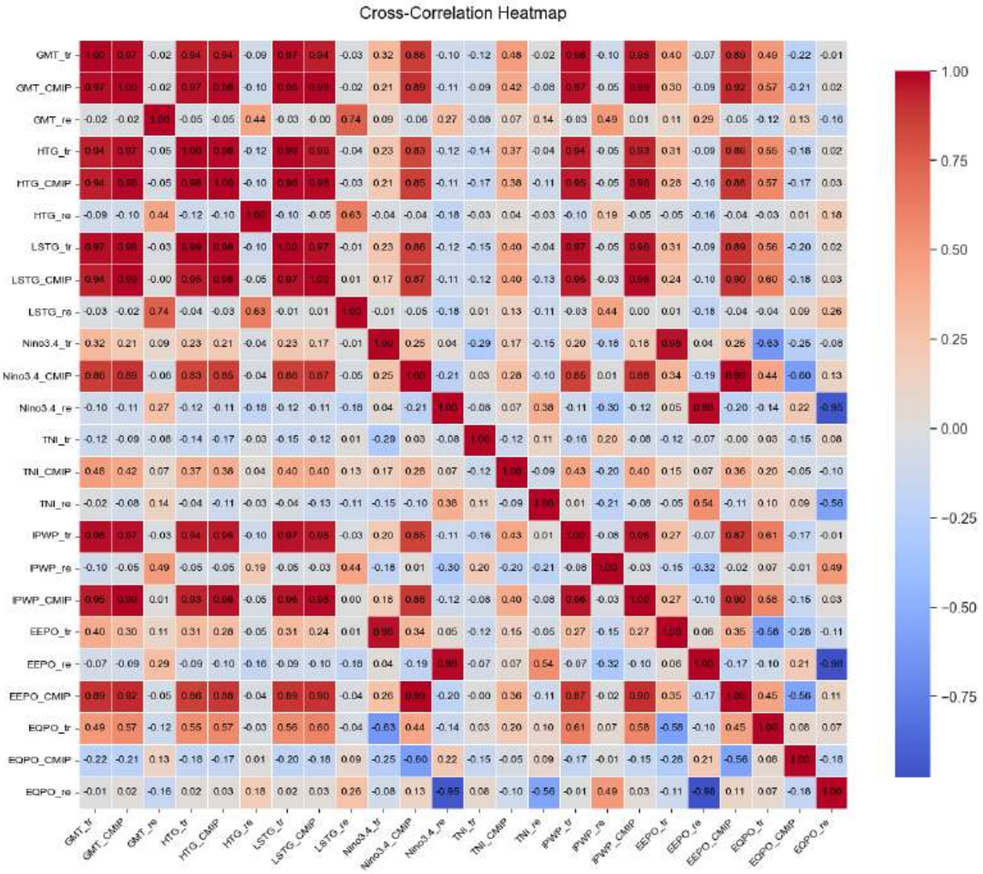

Supplementary Figure 6 Cross-correlation between trend (tr) and residual (re) components (extracted with LOESS; see the Data and Methods section in the main text) of time series for different climate variables from ERA5 and CMIP6 MME. GMT denotes global mean temperature, HTG the interhemispheric thermal gradient, LSTG the land–sea thermal gradient, IPWP the Indo-Pacific warm pool SST, EEPO the equatorial eastern Pacific SST, and EQPO the equatorial Pacific SST gradient (IPWP-EEPO). Full definitions of all variables are provided in the Data and Methods section in the main manuscript.

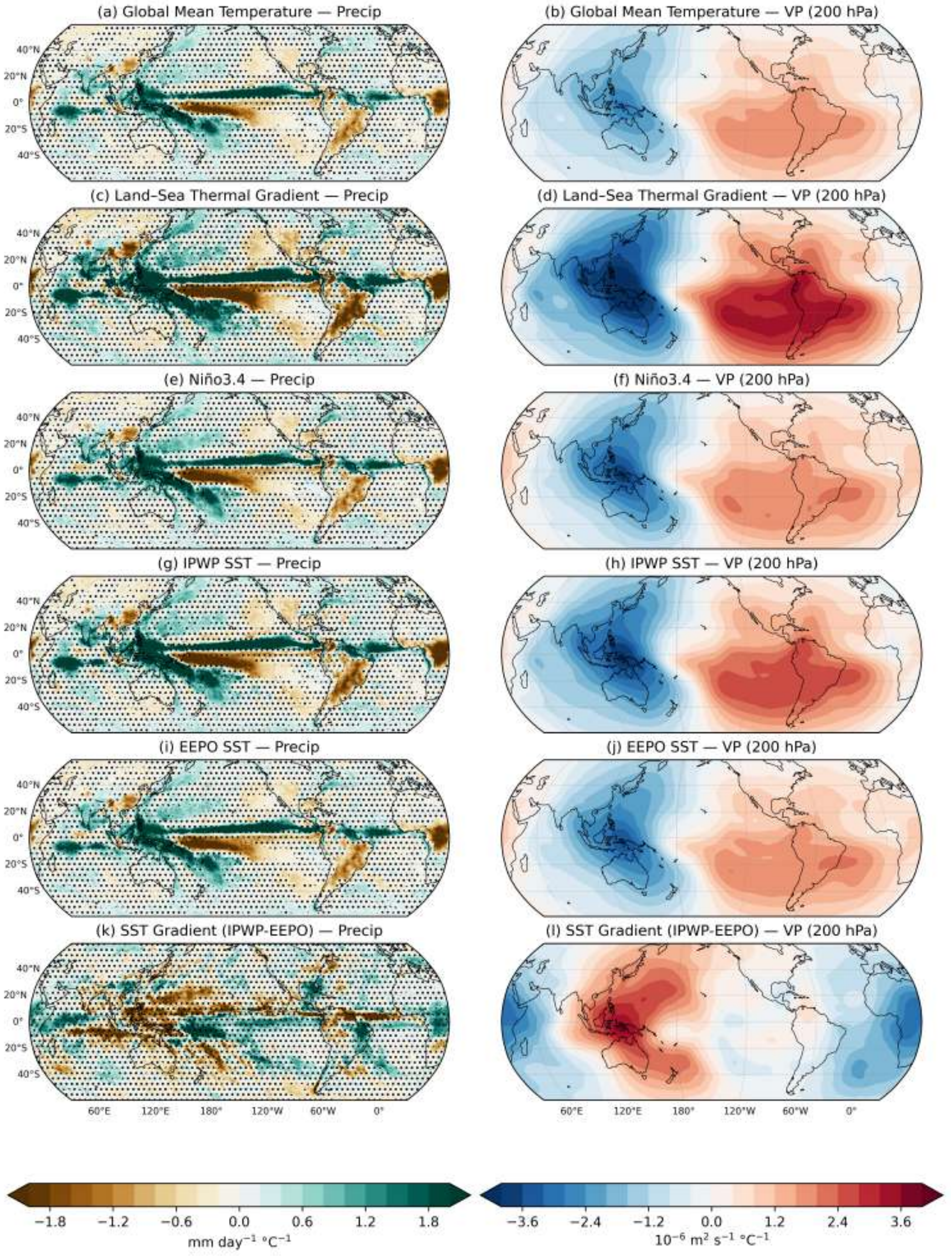

Supplementary Figure 7 Spatial regressions of annual anomalies (1979–2024). Left column: precipitation (mm day^{-1} °C^{-1}); right column: 200-hPa velocity potential (10^{-6} m^2 s^{-1} °C^{-1}), both derived from ERA5. Fields are regressed onto six climate indices derived from the CMIP6 MME: (a,b) GMT, (c,d) land–sea thermal gradient, (e,f) Niño 3.4, (g,h) IPWP SST, (i,j) EEPO SST, and (k,l) equatorial Pacific SST gradient. Colours show regression coefficients; stippling marks grid points where the coefficient is not significant at 90% confidence level.

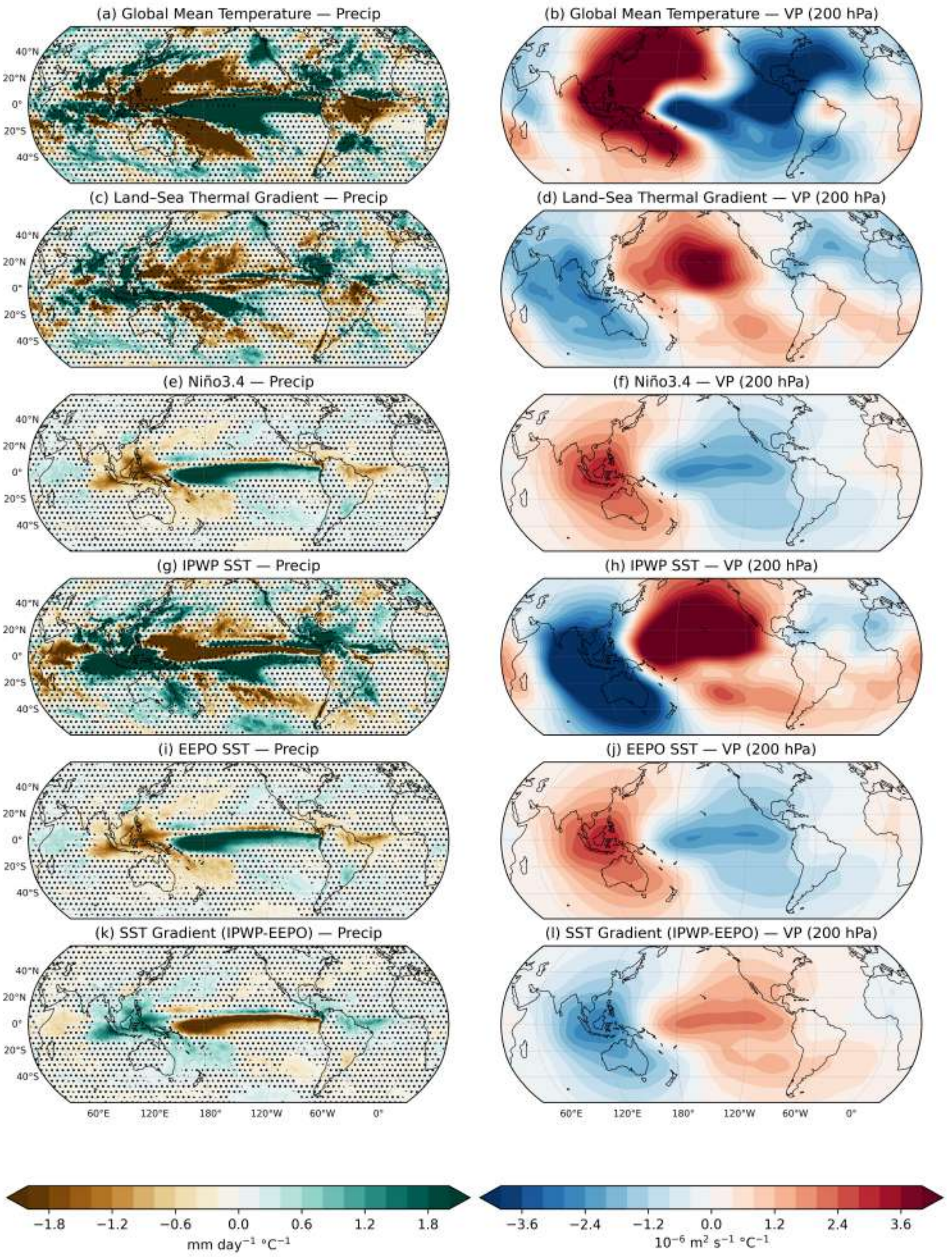

Supplementary Figure 8 Spatial regressions of annual anomalies (1979–2024). Left column: precipitation (mm day^{-1} °C^{-1}); right column: 200-hPa velocity potential (10^{-6} m^2 s^{-1} °C^{-1}), both derived from ERA5. Fields are regressed onto the residual component of the six temperature-related indices derived from the ERA5: (a,b) GMT, (c,d) land–sea thermal gradient, (e,f) Niño 3.4, (g,h) IPWP SST, (i,j) EEPO SST, and (k,l) equatorial Pacific SST gradient (IPWP-EEPO). Colours show regression coefficients; stippling marks grid points where the coefficient is not significant at 90% confidence level.

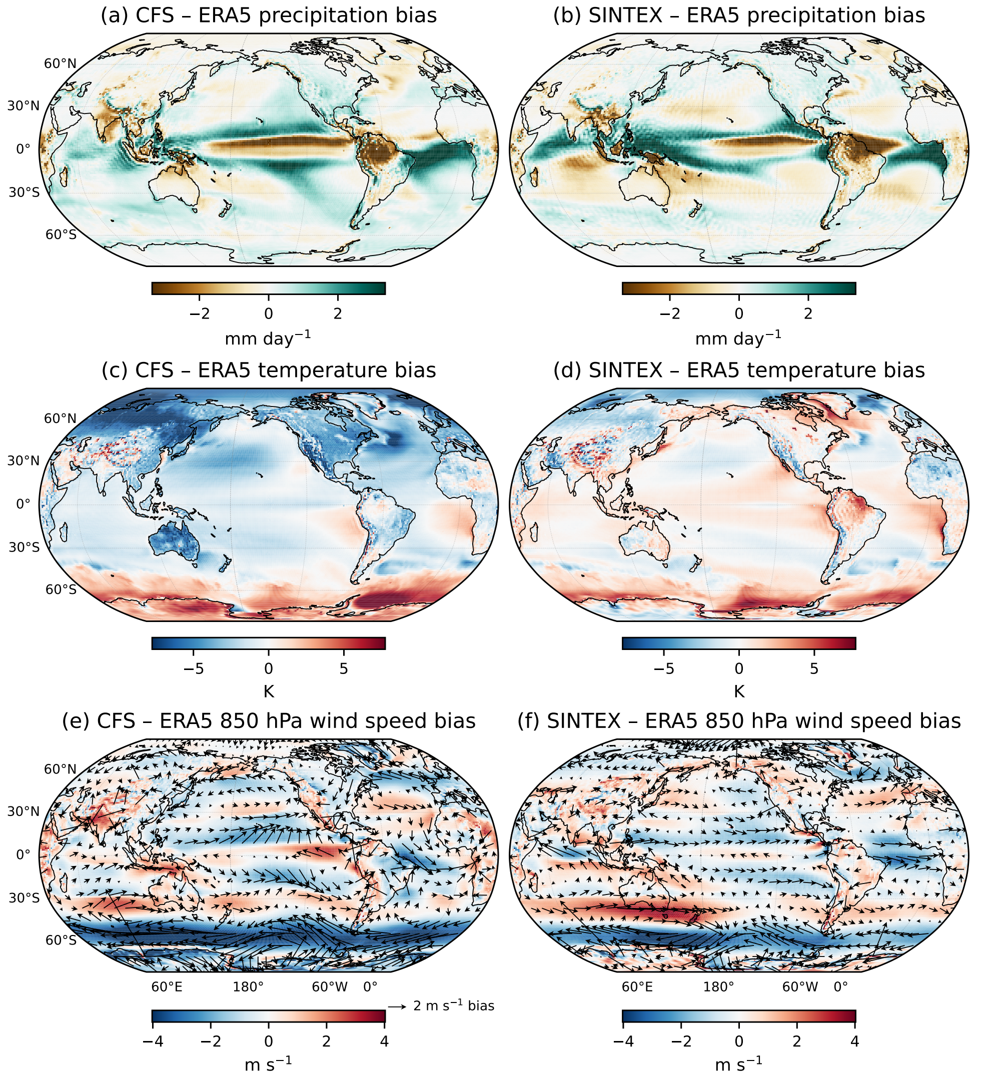

Supplementary Figure 9 Climatological biases of the CFS (left) and SINTEX (right) models used for sensitivity experiments: (a,b) precipitation (mm/day), and (c,d) surface temperature (°C), (e,f) 850-hPa wind vectors (arrows) and speed (shading; m/s). The biases are calculated using the climatological data (1979-2024) from ERA5.

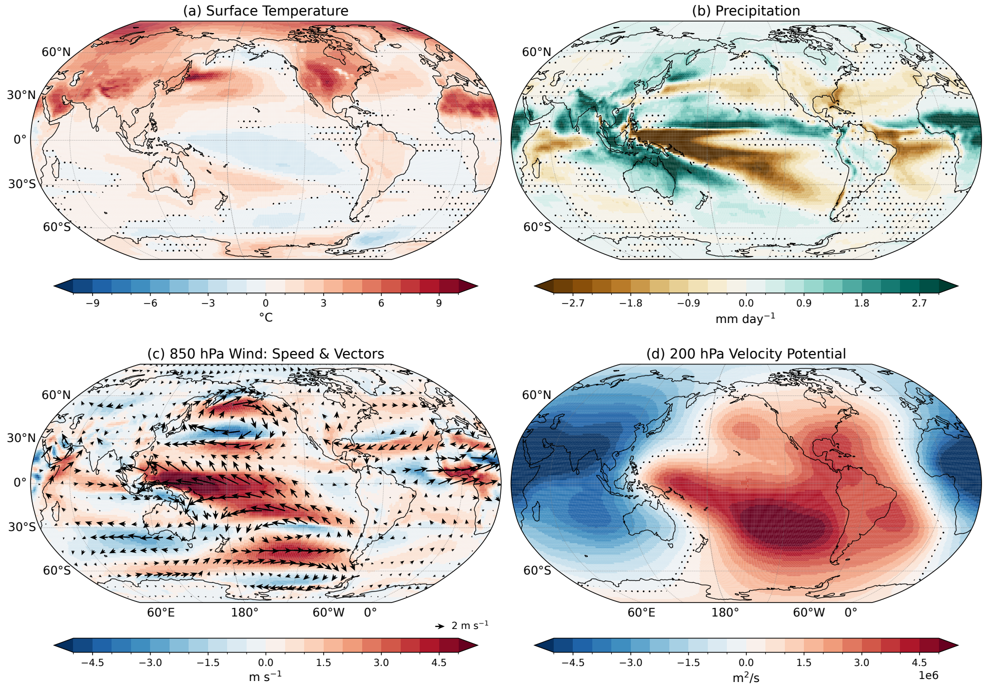

Supplementary Figure 10 Climate model sensitivity to enhanced land–sea thermal contrast. Results are from a coupled experiment using the CFS model, in which the snow-free background land surface albedo is set to zero, maximizing land surface warming relative to the oceans. Panels show the difference between the sensitivity experiment and the MODIS-ctrl simulation for (a) surface temperature (°C), (b) precipitation (mm/day), (c) 850 hPa wind speed (m s^{-1}) with vectors indicating wind direction, and (d) 200 hPa velocity potential (m^2 s^{-1}). Dots represent regions with statistically insignificant differences at 90% confidence level according to the Welch's two-sample t-test. The wind vectors in panels (c) are plotted only if at least one component of the wind difference is statistically significant at 90% confidence level.

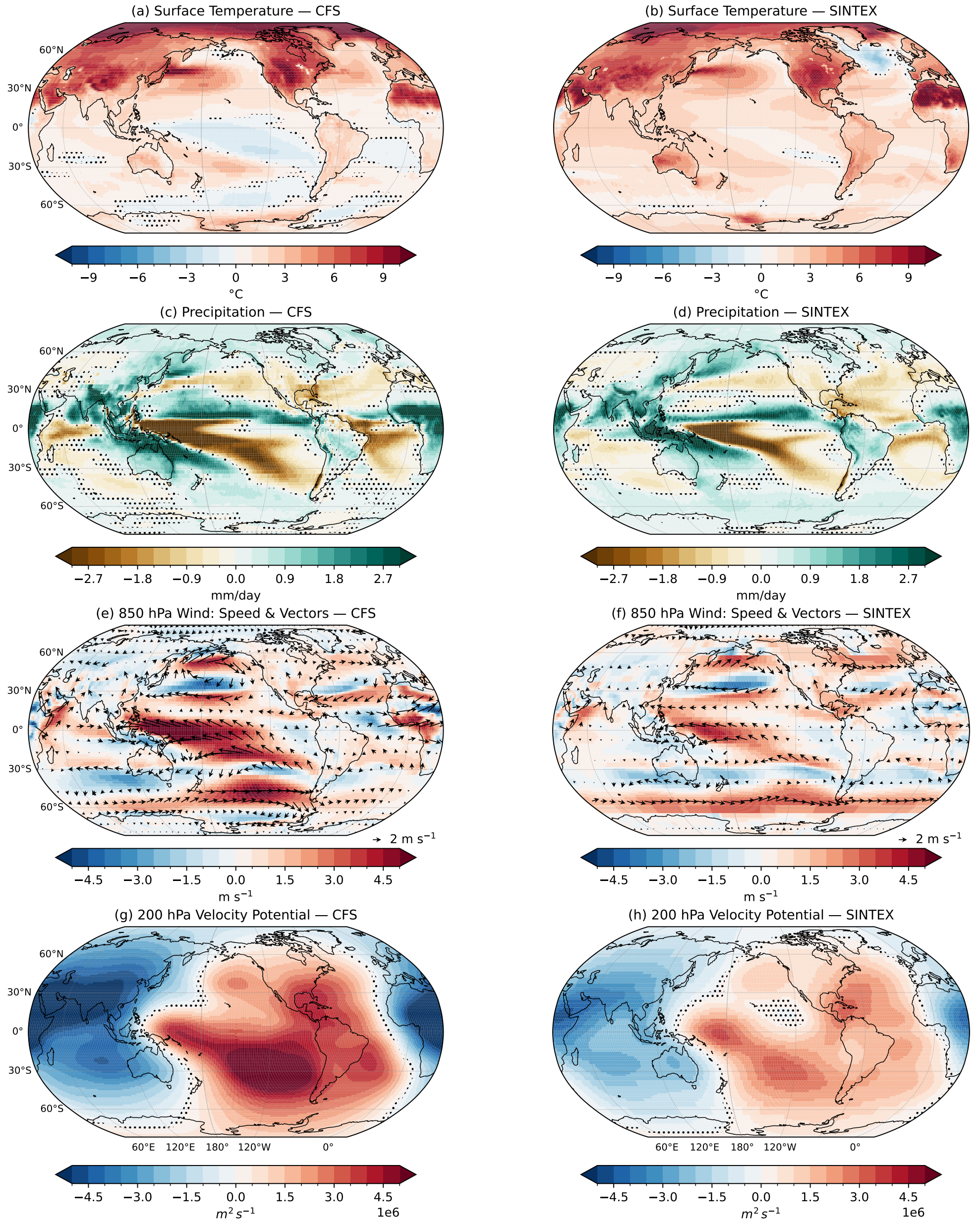

Supplementary Figure 11 Climate model sensitivity to enhanced land–sea thermal contrast. Results are from coupled experiments using CFS (left column) and SINTEX (right column) coupled models, in which the snow-free background land surface albedo is set to zero, maximizing land surface warming relative to the oceans. Panels show the difference between the sensitivity experiments and the control simulations for each model (with standard, unchanged albedo) for (a,b) surface temperature (°C), (c,d) precipitation (mm/day), (e,f) 850 hPa wind speed (m s^{-1}) with vectors indicating wind direction, and (g,h) 200 hPa velocity potential (m^2 s^{-1}). Dots represent regions with statistically insignificant differences at 90% confidence level according to the Welch's two-sample t-test. The wind vectors in panels (e) and (f) are plotted only if at least one component of the wind difference is statistically significant at 90% confidence level.

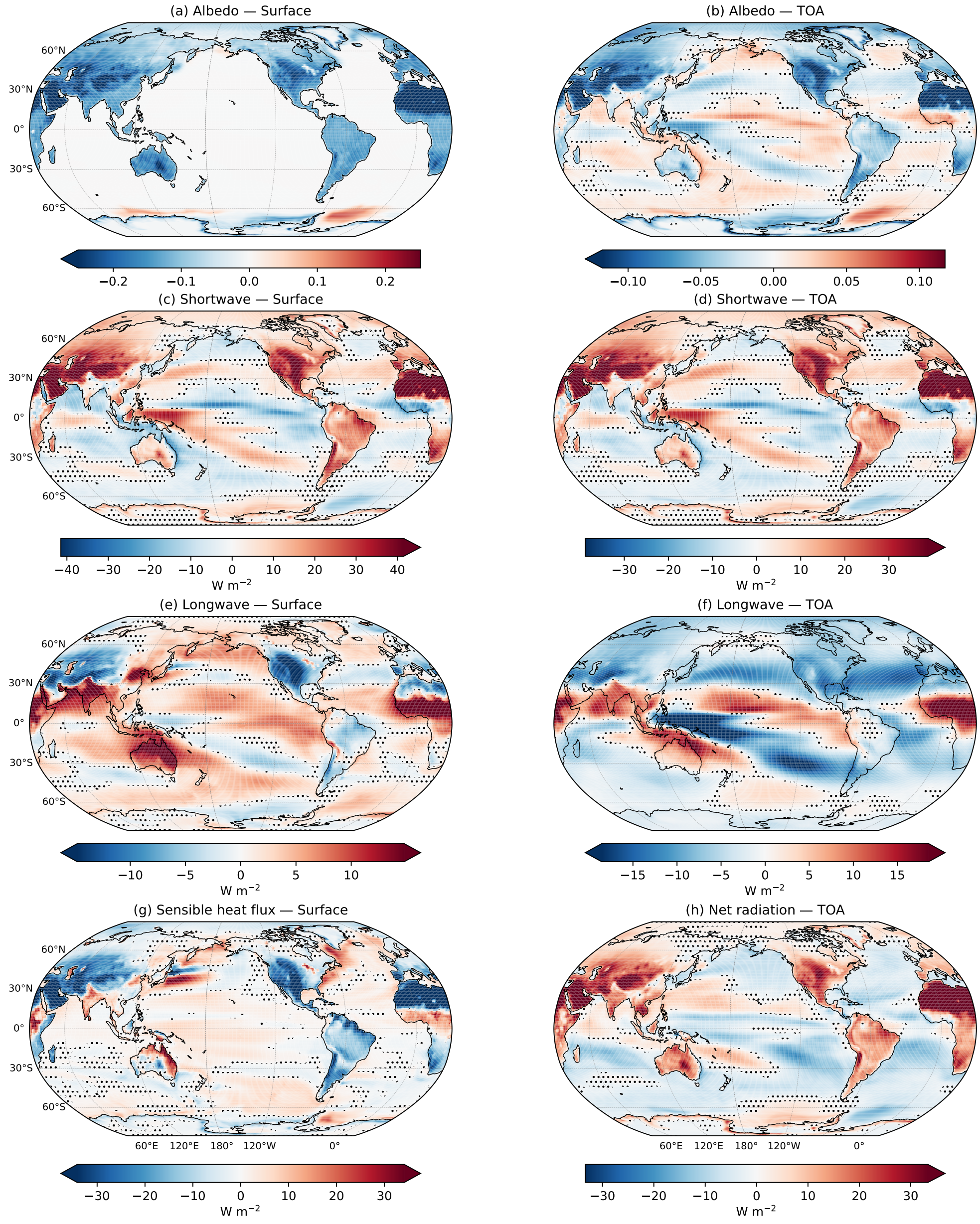

Supplementary Figure 12 Climate model sensitivity to enhanced land–sea thermal contrast. Spatial patterns of the response to an imposed increase in land–sea thermal contrast, obtained from a coupled sensitivity experiment with the CFS model in which the snow-free background land surface albedo is set to zero. Shown are the differences between the sensitivity experiment and the MODIS-ctrl simulation. Left panels show surface responses, and right panels show TOA responses for (a,b) albedo, (c,d) shortwave radiation, (e,f) longwave radiation, (g) sensible heat flux at the surface, and (h) net TOA radiation (units: W m-^{2} for radiation and sensible heat fluxes). Note that downward (upward) fluxes are positive (negative). Stippling indicates regions where differences are not statistically significant at the 90% confidence level based on Welch's two-sample t-test.

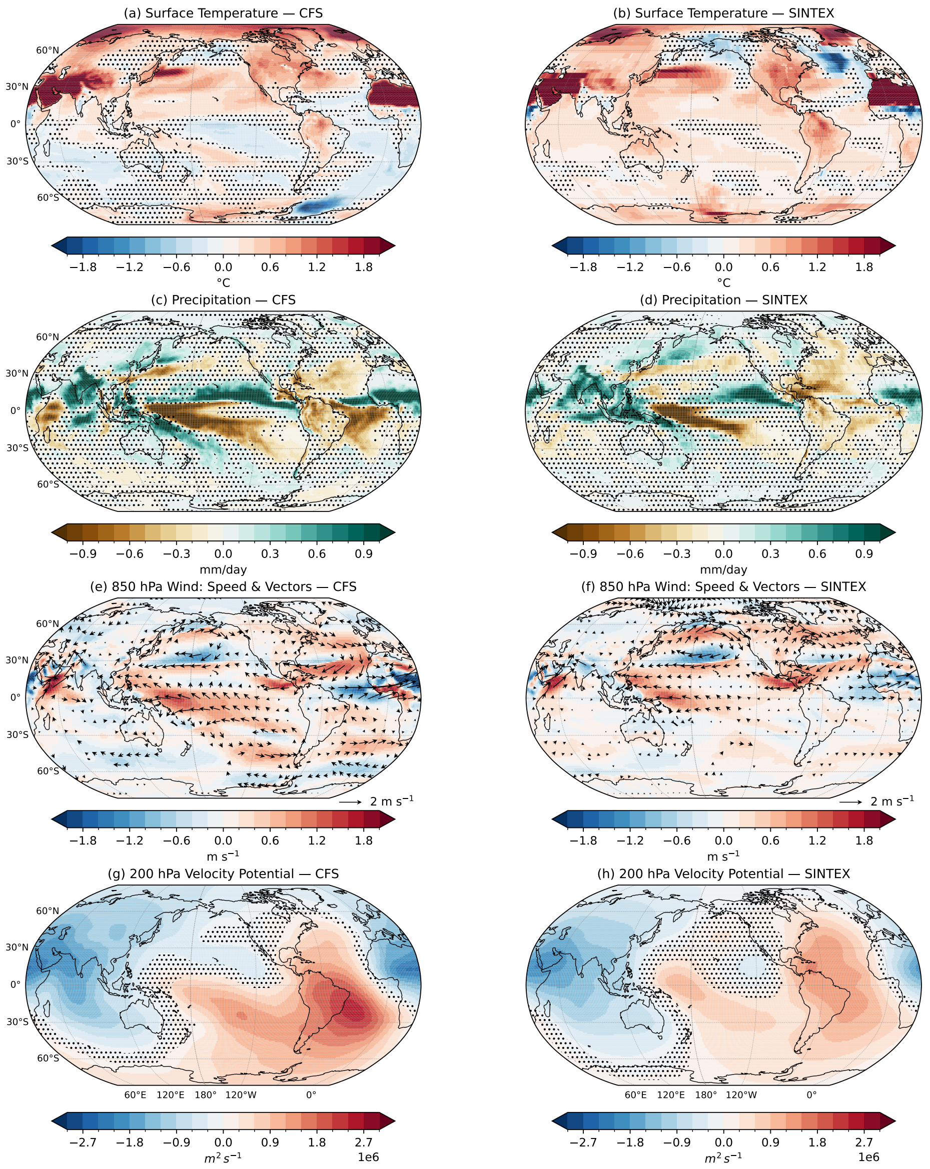

Supplementary Figure 13 Climate model sensitivity to enhanced warming over the arid regions of the Northern Hemisphere. Results are from coupled experiments using CFS (left column) and SINTEX (right column) coupled models, in which the snow-free background land surface albedo is decreased by 20% over the Sahara, Arabia, and Middle East deserts (land regions over 15°–40°N and 20°W–75°E). Panels show the difference between the sensitivity experiments and the MODIS-ctrl simulations for each model (with upgraded snow-free land background albedo with MODIS data) for (a,b) surface temperature (°C), (c,d) precipitation (mm/day), (e,f) 850 hPa wind speed (m s^{-1}) with vectors indicating wind direction, and (g,h) 200 hPa velocity potential (m^2 s^{-1}). Dots represent regions with statistically insignificant differences at 90% confidence level according to the Welch's two-sample t-test. The wind vectors in panels (e) and (f) are plotted only if at least one component of the wind difference is statistically significant at 90% confidence level.

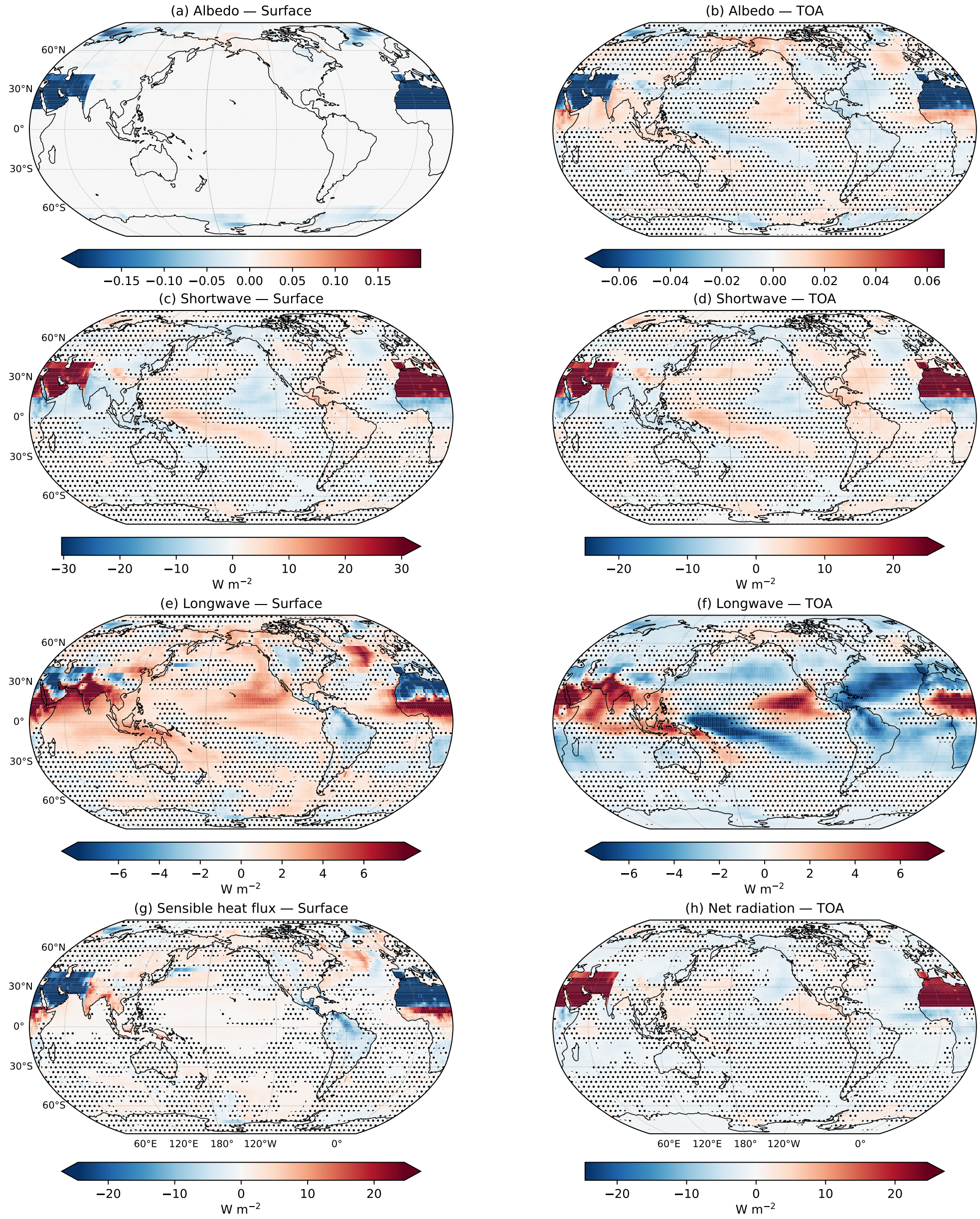

Supplementary Figure 14 Climate model sensitivity to enhanced warming over the arid regions of the Northern Hemisphere. Results are from an experiment using the SINTEX coupled model, in which the snow-free background land surface albedo is decreased by 20% over the Sahara, Arabia, and Middle East deserts (land regions over 15°–40°N and 20°W–75°E). Shown are the differences between the sensitivity experiment and the MODIS-ctrl simulation. Left panels show surface responses, and right panels show TOA responses for (a,b) albedo, (c,d) shortwave radiation, (e,f) longwave radiation, (g) sensible heat flux at the surface, and (h) net TOA radiation (units: W m-$^{\mathbf{2}}$ for radiation and sensible heat fluxes). Note that downward (upward) fluxes are positive (negative). Stippling indicates regions where differences are not statistically significant at the 90% confidence level based on Welch's two-sample t-test.

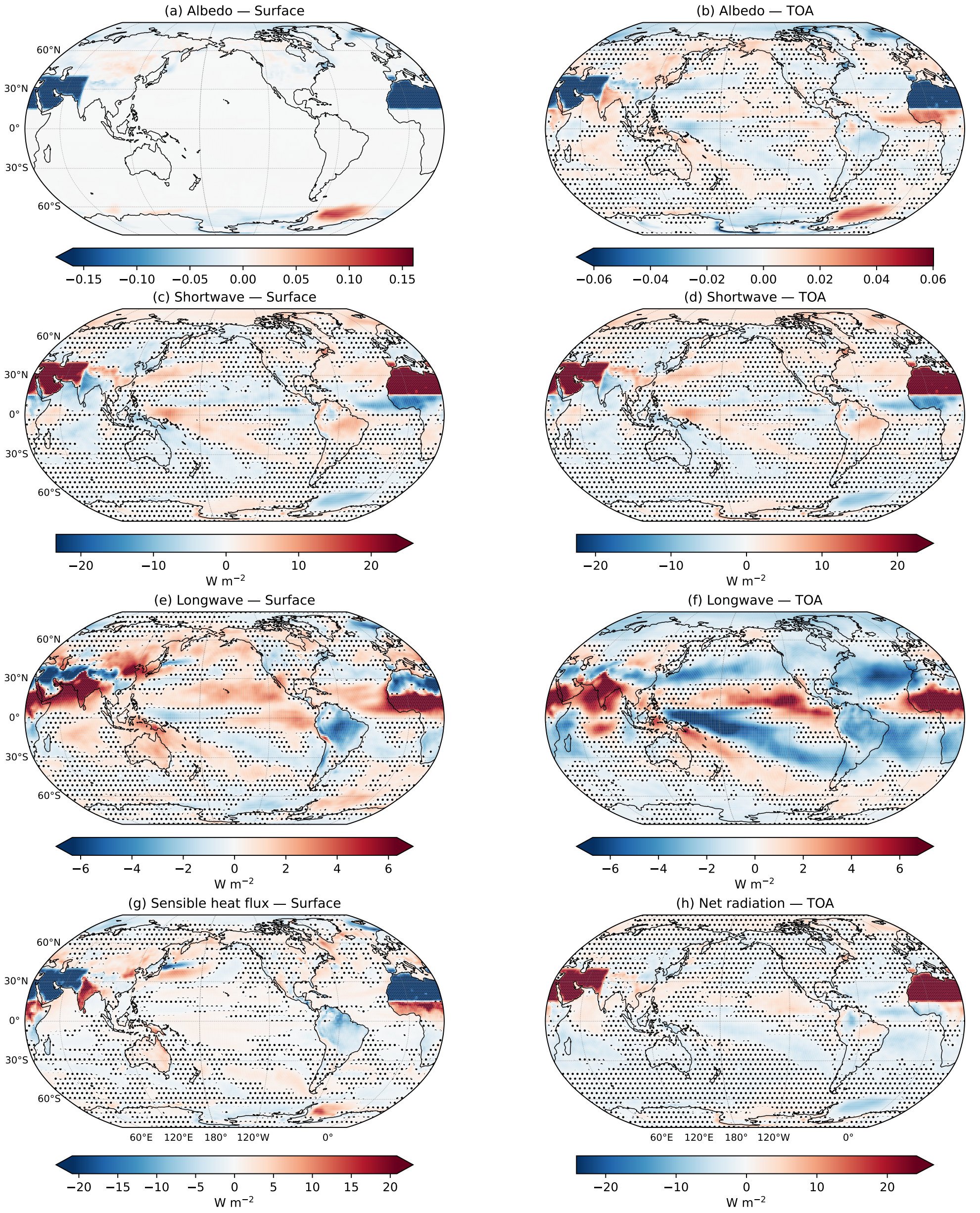

Supplementary Figure 15 Climate model sensitivity to enhanced warming over the arid regions of the Northern Hemisphere. Results are from an experiment using the CFS coupled model, in which the snow-free background land albedo is decreased by 20% over the Sahara, Arabia, and Middle East deserts (land regions over 15°–40°N and 20°W–75°E). Shown are the differences between the sensitivity experiment and the MODIS-ctrl simulation. Left panels show surface responses, and right panels show TOA responses for (a,b) albedo, (c,d) shortwave radiation, (e,f) longwave radiation, (g) sensible heat flux at the surface, and (h) net TOA radiation (units: W m^{-2} for radiation and sensible heat fluxes). Note that downward (upward) fluxes are positive (negative). Stippling indicates regions where differences are not statistically significant at the 90% confidence level based on Welch's two-sample t-test.

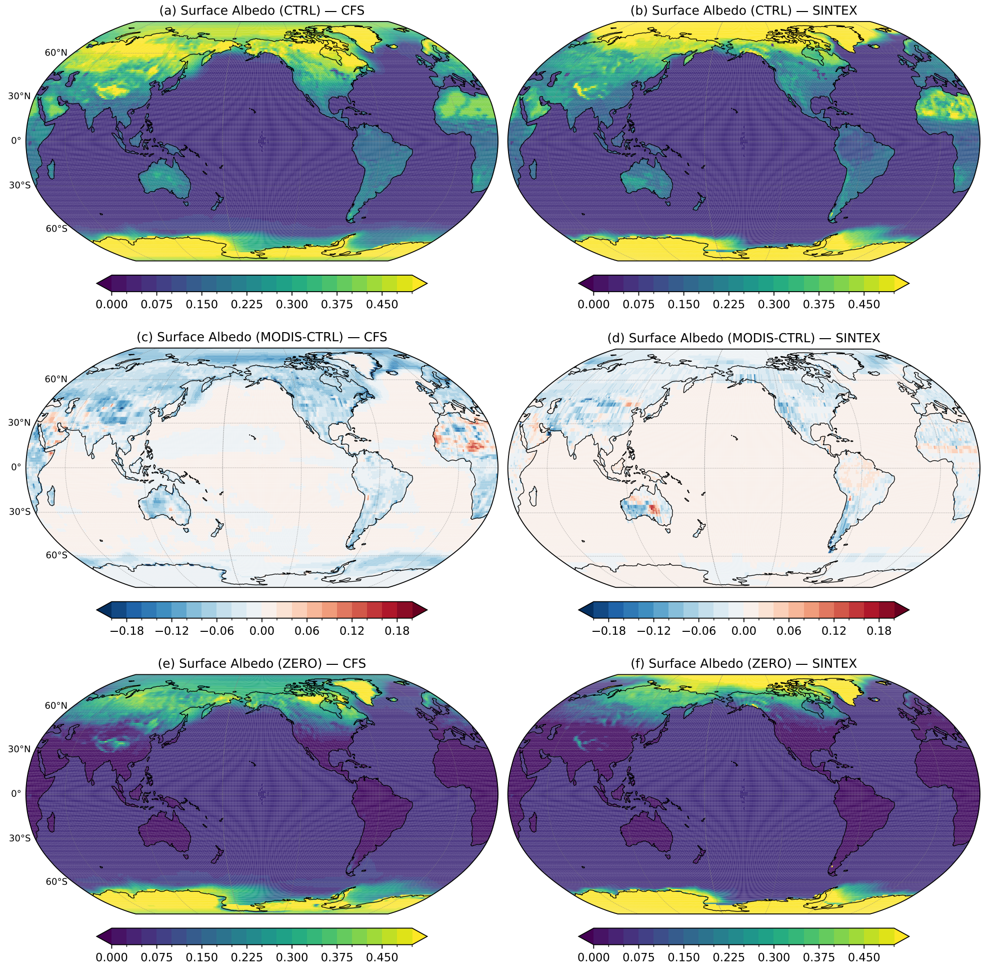

Supplementary Figure 16 Global distribution of surface albedo climatology from CFS and SINTEX experiments. The first row shows the ctrl simulation climatology of surface albedo (CTRL), the second row shows the differences between the MODIS-ctrl and ctrl experiments's climatologies of surface albedo (MODIS - CTRL), and the third row shows the surface albedo climatologies in the zero-albedo experiments (ZERO). The left column corresponds to the CFS model and the right column to the SINTEX model.

References

[1] Adler, R.F., Huffman, G.J., Chang, A., Ferraro, R., Xie, P.-P., Janowiak, J., Rudolf, B., Schneider, U., Curtis, S., Bolvin, D., Gruber, A., Susskind, J., Arkin, P., Nelkin, E.: The version-2 global precipitation climatology project (gpcp) monthly precipitation analysis (1979–present). Journal of Hydrometeorology **4**(6), 1147–1167 (2003) https://doi.org/10.1175/1525-7541(2003)004⟨1147:tvgpcp⟩2.0.co;2

[2] Rohde, R.A., Hausfather, Z.: The berkeley earth land/ocean temperature record. Earth System Science Data **12**(4), 3469–3479 (2020) https://doi.org/10.5194/essd-12-3469-2020

[3] Huang, B., Liu, C., Banzon, V., Freeman, E., Graham, G., Hankins, B., Smith, T., Zhang, H.-M.: Improvements of the daily optimum interpolation sea surface temperature (doisst) version 2.1. Journal of Climate **34**(8), 2923–2939 (2021) https://doi.org/10.1175/jcli-d-20-0166.1

[4] Kanamitsu, M., Ebisuzaki, W., Woollen, J., Yang, S.-K., Hnilo, J.J., Fiorino, M., Potter, G.L.: Ncep–doe amip-ii reanalysis (r-2). Bulletin of the American Meteorological Society **83**(11), 1631–1644 (2002) https://doi.org/10.1175/bams-83-11-1631

[5] Dawson, A.: Windspharm: A high-level library for global wind field computations using spherical harmonics. Journal of Open Research Software **4**(1), 31 (2016) https://doi.org/10.5334/jors.129

[6] Terray, P., Sooraj, K.P., Masson, S., Krishna, R.P.M., Samson, G., Prajeesh, A.G.: Towards a realistic simulation of boreal summer tropical rainfall climatology in state-of-the-art coupled models: role of the background snow-free land albedo. Climate Dynamics **50**(9–10), 3413–3439 (2018) https://doi.org/10.1007/s00382-017-3812-9

[7] Xiang, B., Zhao, M., Held, I.M., Golaz, J.: Predicting the severity of spurious "double itcz" problem in cmip5 coupled models from amip simulations. Geophysical Research Letters **44**(3), 1520–1527 (2017) https://doi.org/10.1002/2016gl071992

[8] Tian, B., Dong, X.: The double-itcz bias in cmip3, cmip5, and cmip6 models based on annual mean precipitation. Geophysical Research Letters **47**(8) (2020) https://doi.org/10.1029/2020gl087232

[9] Fiedler, S., Crueger, T., D'Agostino, R., Peters, K., Becker, T., Leutwyler, D., Paccini, L., Burdanowitz, J., Buehler, S.A., Cortes, A.U., Dauhut, T., Dommenget, D., Fraedrich, K., Jungandreas, L., Maher, N., Naumann, A.K., Rugenstein, M., Sakradzija, M., Schmidt, H., Sielmann, F., Stephan, C., Timmreck, C., Zhu, X., Stevens, B.: Simulated tropical precipitation assessed across three major

phases of the coupled model intercomparison project (cmip). Monthly Weather Review **148**(9), 3653–3680 (2020) https://doi.org/10.1175/mwr-d-19-0404.1

[10] Bracegirdle, T.J., Holmes, C.R., Hosking, J.S., Marshall, G.J., Osman, M., Patterson, M., Rackow, T.: Improvements in circumpolar southern hemisphere extratropical atmospheric circulation in cmip6 compared to cmip5. Earth and Space Science **7**(6) (2020) https://doi.org/10.1029/2019ea001065

[11] Chadwick, R., Boutle, I., Martin, G.: Spatial patterns of precipitation change in cmip5: Why the rich do not get richer in the tropics. Journal of Climate **26**(11), 3803–3822 (2013) https://doi.org/10.1175/jcli-d-12-00543.1
